# Clean Hands

**Published:** 1916-11

**Authors:** 


### Abstracts and Selections.

### Clean Hands.

Disease germs lead a hand-to-mouth existence. If the human race would learn to keep the unwashed hand away from the mouth many human diseases would be greatly diminished. We handle infectious matter more or less constantly and we continually carry the hands to the mouth. If the hand has recently been in contact with infectious matter the germs of disease may in this way be introduced into the body. Many persons wet their fingers with saliva before counting money, turning the pages of a book, or performing similar acts. In this case the process is reversed, the infection being carried to the object handled, there to await carriage to the mouth of some other careless person. In view of these facts the United States Public Health Service has formulated the following simple rules of personal hygiene and recommends their adoption by every person in the United States:

WASH THE HANDS IMMEDIATELY

Before eating,
Before handling, preparing or serving food,
After using the toilet,
After attending the sick, and
After handling anything dirty.

